# Loss of Pcgf5 Affects Global H2A Monoubiquitination but Not the Function of Hematopoietic Stem and Progenitor Cells

**DOI:** 10.1371/journal.pone.0154561

**Published:** 2016-05-02

**Authors:** Sha Si, Yaeko Nakajima-Takagi, Kazumasa Aoyama, Motohiko Oshima, Atsunori Saraya, Hiroki Sugishita, Manabu Nakayama, Tomoyuki Ishikura, Haruhiko Koseki, Atsushi Iwama

**Affiliations:** 1 Department of Cellular and Molecular Medicine, Graduate School of Medicine, Chiba University, Chiba, Japan; 2 Chromosome Engineering Team, Department of Technology Development, Kazusa DNA Research Institute, Kisarazu, Japan; 3 Laboratory for Developmental Genetics, RIKEN Center for Integrative Medical Sciences (IMS), Yokohama, Japan; B.C. Cancer Agency, CANADA

## Abstract

Polycomb-group RING finger proteins (Pcgf1-Pcgf6) are components of Polycomb repressive complex 1 (PRC1)-related complexes that catalyze monoubiquitination of histone H2A at lysine 119 (H2AK119ub1), an epigenetic mark associated with repression of genes. Pcgf5 has been characterized as a component of PRC1.5, one of the non-canonical PRC1, consisting of Ring1a/b, Rybp/Yaf2 and Auts2. However, the biological functions of Pcgf5 have not yet been identified. Here we analyzed the impact of the deletion of *Pcgf5* specifically in hematopoietic stem and progenitor cells (HSPCs). *Pcgf5* is expressed preferentially in hematopoietic stem cells (HSCs) and multipotent progenitors (MPPs) compared with committed myeloid progenitors and differentiated cells. We transplanted bone marrow (BM) cells from *Rosa*::*Cre-ERT* control and *Cre-ERT;Pcgf5*^*fl/fl*^ mice into lethally irradiated recipient mice. At 4 weeks post-transplantation, we deleted *Pcgf5* by injecting tamoxifen, however, no obvious changes in hematopoiesis were detected including the number of HSPCs during a long-term observation period following the deletion. Competitive BM repopulating assays revealed normal repopulating capacity of *Pcgf5*-deficient HSCs. Nevertheless, *Pcgf5*-deficient HSPCs showed a significant reduction in H2AK119ub1 levels compared with the control. ChIP-sequence analysis confirmed the reduction in H2AK119ub1 levels, but revealed no significant association of changes in H2AK119ub1 levels with gene expression levels. Our findings demonstrate that Pcgf5-containing PRC1 functions as a histone modifier *in vivo*, but its role in HSPCs is limited and can be compensated by other PRC1-related complexes in HSPCs.

## Introduction

Epigenetic regulation has a critical role not only in normal hematopoiesis but also in hematological malignancies [1–3]. Polycomb-group (PcG) proteins are key regulators of the epigenetic machinery that establish and maintain reversible gene silencing. PcG proteins form various polycomb repressive complexes (PRC). The PRC1 and PRC2 complexes possess H2AK119 ubiquitin ligase activity and H3K27 methyltransferase activity, respectively. Six PRC1-related complexes containing distinct Polycomb-group RING finger proteins (Pcgf1-Pcgf6) have been identified [4,5].

PcG complexes have been well characterized as general regulators of stem cells [6,7]. Pcgf4/Bmi1, a component of canonical PRC1 (PRC1.4), plays a central role in the maintenance of self-renewal and multipotency of hematopoietic stem cells (HSCs) by targeting *p16*^*Ink4a*^ and *p19*^*Arf*^ tumor suppressor genes and developmental regulator genes [8–10]. PRC2 complex has a well-established role in the maintenance of HSCs [11,12]. In addition to their role in stem cells, PcG proteins also function in tumor-initiating cells, where they are often deregulated, leading to the promotion of tumorigenesis. Thus, PcG genes act as both oncogenes as well as tumor suppressor genes depending on cell type [3, 13–15].

PRC1.5 is one of the emerging variant PRC1 complexes, and consists of Ring1a/b, Pcgf5, Rybp/Yaf2 and Auts2. Pcgf5 and Auts2 are components unique to PRC1.5. Of interest, Auts2 has been shown to render PRC1 capable of activating transcription by recruiting casein kinase 2 and p300 in developing neuronal cells [16]. In contrast, Pcgf5 has been demonstrated to contribute to H2AK119ub1-dependent recruitment of PRC2 and H3K27me3 modification in a manner similar to other variant PRC1 complexes, Pcgf1 and Pcgf3, in a *de novo* targeting assay in mouse embryonic stem cells (ESCs) [17]. However, its role *in vivo* remains to be investigated.

In this study, we analyzed the role of Pcgf5 in hematopoietic stem and progenitor cells (HSPCs). Using a *Pcgf5* conditional knockout mouse model and comprehensive expression and epigenetic analyses, we demonstrate that Pcgf5 regulates global H2A monoubiquitylation but is dispensable for hematopoietic stem and progenitor cells

## Materials and Methods

### Ethics Statement

Experiments using mice were performed in accordance with institutional guidelines of the Graduate School of Medicine, Chiba University. This study was approved by the Institutional Review Committees of Chiba University (approval numbers 24–64 and 27–213).

### Mice and gene targeting of *Pcgf5*

The conditional *Pcgf5* allele (*Pcgf5*^*fl*^), which contains LoxP sites flanking *Pcgf5* exon 2 containing the first ATG, was generated by homologous recombination using R1 embryonic stem (ES) cells according to the conventional protocol. *Pcgf5*^*fl/+*^ mice were backcrossed to the C57BL/6 background more than 5 times and crossed with *Rosa*::*Cre-ERT2* mice (TaconicArtemis GmbH). To induce Cre activity, mice were injected with 100 μl of tamoxifen dissolved in corn oil at a concentration of 10 mg/ml intraperitoneally once a day for 5 consecutive days 1 month after transplantation. C57BL/6 mice congenic for the Ly5 locus (CD45.1) were purchased from Sankyo Lab Service.

### Locus-specific genotyping of *Pcgf5*

We performed genotyping of *Pcgf5* allele using the following primers:

5’-GACCCTGAAGGAGTTGGCTCG-3’ and 5’- TGGCCTTGGTACACATATAGC-3’ for *flox* allele, and 5’-TGTTTACAGAGAGGAAGCGCC-3’ and 5’-TGGCCTTGGTACACATATAGC-3’ for *delta* allele.

### Bone marrow transplantation

Bone marrow (BM) cells from test mice (CD45.2) were injected via the tail veins of 8-week-old CD45.1 recipients lethally irradiated at a dose of 9.5 Gy with or without competitor BM cells from 8-week-old CD45.1 congenic mice. For secondary transplantation, 5 × 10^6^ BM cells pooled from the primary recipient mice at 4 months post-transplantation were injected into 8-week-old CD45.1 mice (secondary recipient mice) irradiated at a dose of 9.5 Gy without competitor cells.

### Purification of hematopoietic cells and flow cytometric analysis

BM mononuclear cells were incubated with APC-conjugated anti-c-Kit antibody followed by anti-APC MicroBeads (Miltenyi Biotec). c-Kit^+^ cells were immunomagnetically enriched by passing through an LS column (Miltenyi Biotec). Purified c-Kit^+^ cells were then stained with a mixture of biotin-conjugated mAbs against lineage markers, including Gr-1, Mac-1, interleukin (IL)-7Rα, B220, CD4, CD8, and Ter119, and FITC-conjugated anti-CD34, PE-conjugated anti-FcγRII/III, PE-Cy7-conjugated anti-Sca-1, and APC-conjugated anti-c-Kit antibodies. Biotinylated antibodies were detected with APC-Cy7-conjugated streptavidin. CD45.1 and CD45.2 antibodies were used as additional markers for recipient cells and donor-derived cells, respectively. Flow cytometric analyses were performed using antibodies recognizing the following antigens: CD45.2 (104), CD45.1(A20), Gr-1 (RB6-8C5), CD11b/Mac-1 (M1/70), Ter-119, CD127/IL-7Rα (A7R34, SB/199), B220 (RA3-6B2), CD4 (GK1.5, RM4-5), CD8α (53–6.7), CD117/c-Kit (2B8), Sca-1 (D7), CD135 (A2F10) and CD16/32/FcγRII-III (93). The antibodies were purchased from BD Biosciences, eBioscience, and BioLegend. Dead cells were eliminated by staining with 0.5 μg/ml propidium iodide (Sigma-Aldrich). The data were acquired on a FACS Aria II cell sorter or a Canto II flow cytometer (both BD Biosciences), and analyzed using Flowjo Version 10.0.6 software (TreeStar).

### Chromatin immunoprecipitation (ChIP) assay and ChIP-Sequence analysis

FACS-sorted GMPs from the BM of recipient mice were cross-linked with 0.5% formaldehyde for 2 minutes at 37°C, washed twice with phosphate-buffered saline, suspended in ChIP buffer (10 mM Tris-HCl, pH 8.0, 200 mM NaCl, 1 mM CaCl_2_, 0.5% NP-40, and protease inhibitor cocktail), sonicated 3 times for 5 seconds using Bioruptor (Cosmo Bio), digested by MNase (New England BioLabs) for 40 minutes at 37°C, lysed with radioimmunoprecipitation assay (RIPA) buffer (50mM Tris-HCl, pH 8.0, 150 mM NaCl, 2 mM EDTA, 1% NP-40, 0.1% SDS and 0.5% sodium deoxycholate) and then sonicated 10 times for 5 seconds using Bioruptor (Cosmo Bio). Dynabeads M-280 Sheep anti-Rabbit IgG (Life technologies) blocked with bovine serum albumin was used for collection of chromatin. Before the immunoprecipitation, 20 μl of Dynabeads was incubated with an anti–H3K27me3 antibody (07–449; Millipore) or an anti–monoubiquitinated H2A (H2Aub1; 8240S, Cell Signaling Technology) for 2 hours at 4°C. Chromatin was immunoprecipitated overnight at 4°C with antibody-conjugated Dynabeads. The immunoprecipitates were washed extensively with the following combination of wash buffers: ChIP buffer, ChIP wash buffer (10 mM Tris-HCl, pH 8.0, 500 mM NaCl, 1 mM CaCl_2_, 0.5% NP-40), and TE buffer (10 mM Tris-HCl, pH 8.0, and 1 mM EDTA). Bound chromatin and input DNA were placed in ChIP elution buffer (50 mM Tris-HCl, pH 8.0, 10 mM EDTA and 1% SDS) and reverse cross-linked. Immunoprecipitated DNA and input DNA were treated with RNase A (Sigma-Aldrich) and proteinase K (Roche), and purified with a QIAquick PCR purification kit (Qiagen). Libraries for ChIP-sequene were generated using ThruPLEX DNA-seq Kit (Rubicon genomics).

### RNA-sequence

Total RNA isolation was performed using an RNeasy plus Micro Kit (Qiagen) according to the manufacturer’s instructions. cDNA was synthesized using a SMARTer Ultra Low Input RNA Kit for Sequencing (Clontech). cDNA libraries were generated with 6x10^3^ LSK cells and GMPs using a NEBNext Ultra DNA Library Prep Kit (New England BioLabs) according to the manufacturer’s indications. The RNA-sequence reads were aligned using TopHat 1 (version 2.0.13; with default parameters) and levels of gene expression were quantified using Cufflinks (version 2.2.1).

### RT-PCR

Total RNA was isolated using TRIZOL LS solution (Invitrogen) and reverse-transcribed by the ThermoScript RT-PCR system (Invitrogen) with an oligo-dT primer. Quantitative RT-PCR (RT-qPCR) was performed with a StepOnePlus Real-Time PCR System (Life Technologies) using FastStart Universal Probe Master (Roche) and the indicated combinations of Universal Probe Library (Roche) and primers listed below. *Hprt* expression was used to calculate relative expression levels. Probe numbers and primer sequences were: Probe #26, 5’-AGATGGCGACTAAGAG GAGAAA-3’ and 5’-ACAAATAGTGCAGGATTCATTCAG-3’ for *Pcgf5*; and probe #95, 5’-TCCTCCTCAGACCGCTTTT-3’ and 5’- CCTGGTTCATCATCGCTAATC-3’ for *Hprt*. To amplify truncated *Pcgf5* mRNA, primers directed to exon 1 and exon 5/6 junction were used: 5’-GGCGCTGTTTCTCTTTCGC-3’ for exon 1 and 5’-CTTCGAAATATCATCTTGCCC-3’ for exon 5/6 junction.

### Immunoprecipitation and Western blot analysis for histone modification

*Pcgf5*^*fl/fl*^*;Rosa*::*Cre-ERT2* ES cells were derived from blastocysts. Conditional deletion of *Pcgf5* was carried out by the addition of 800 nM 4-hydroxytamoxifen for 48 h in culture. For collection, ES cells were trypsinized and plated to gelatin-coated dishes for 30 min to remove contaminating feeder cells. The cells were lysed in 0.1% NP-40 lysis buffer (300 mM NaCl) and centrifuged. The resulting supernatants were kept on ice (Solution A). The pellets were resuspended in 0.1% NP-40 lysis buffer (300 mM NaCl) and sonicated using a Bioruptor (Cosmo Bio) (Solution B). The mixtures of Solution A and Solution B were diluted with 0.1% NP-40 lysis buffer (0 mM NaCl) until the final NaCl concentrations reached 150 mM. After centrifugation, the resulting supernatants were used as input lysates for immunoprecipitation, which was performed using an anti-Ring1b antibody (D139-3, MBL). Total lysates were used to detect histone proteins. Proteins were separated by SDS-PAGE, transferred to a PVDF membrane and detected by Western blotting using the following antibodies: anti-Pcgf5 antibody (ab76724, Abcam), anti-H3 (ab1791, Abcam), anti-H3K27me3 (07–449, Millipore), anti-H2A (ab18255, Abcam), and anti-H2AK119ub (8240S, Cell Signaling Technology). The protein bands were detected with enhanced chemiluminescence reagent (Immobilon Western, Millipore). Sequential reprobing of membranes with antibodies was performed after the removal of primary and secondary antibodies from membranes in 0.2M glycine-HCl buffer (pH 2.5) and/or inactivation of HRP by 0.1% NaN3.

### Statistical analysis

Statistical tests were carried out using Graph Pad Prism version 6. Data are shown as the mean ± SD. Statistical significance was taken at values of *p less than .05, ** *p* less than .01, and *** *p* less than .001.

### Accession numbers

RNA-sequence and ChIP-sequence data were deposited in DNA Data Bank of Japan (DDBJ) (accession number DRA004231 and DRA004597).

## Results

### *Pcgf5* is preferentially expressed in hematopoietic stem and progenitor cells

**Fig 1:**
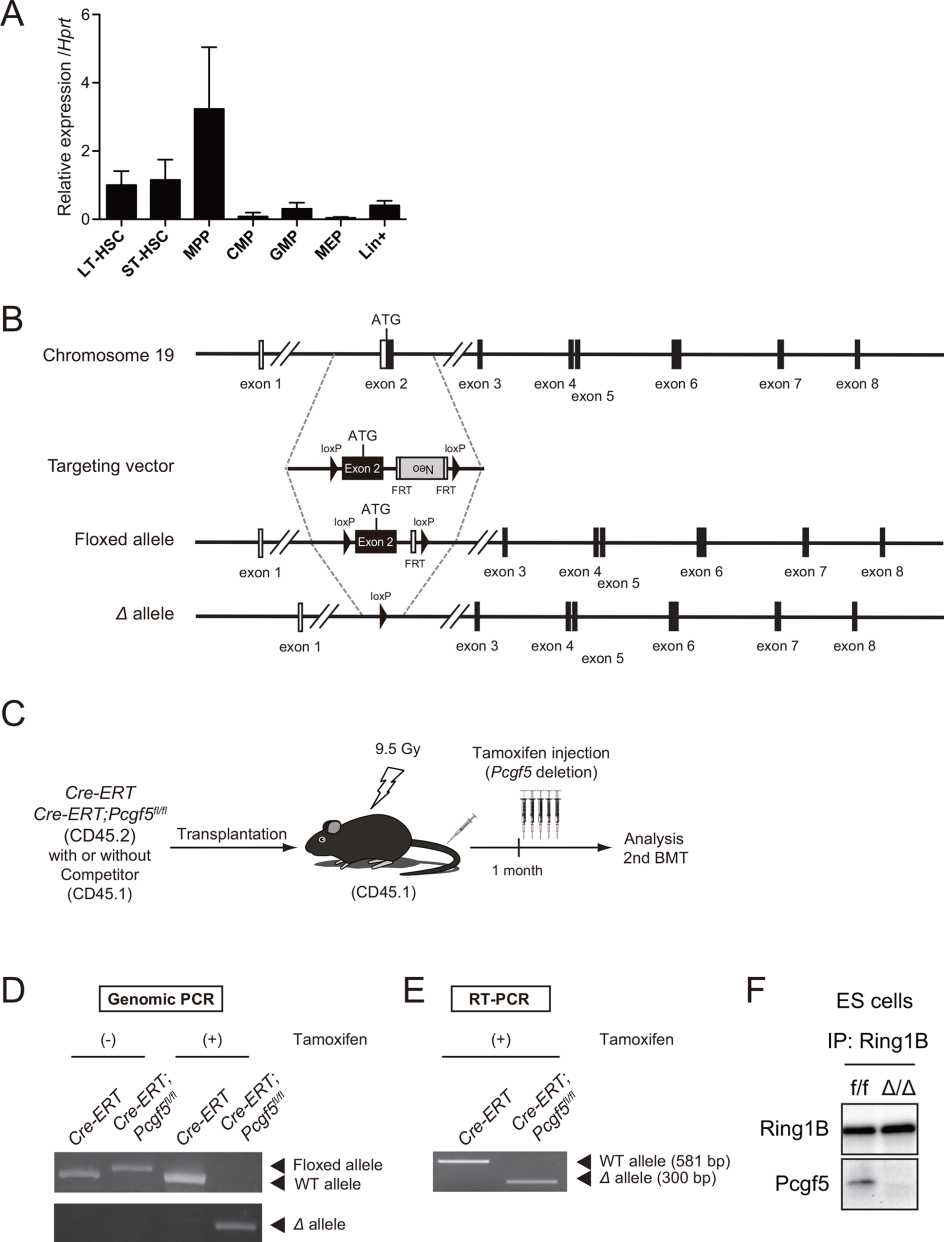
Generation of conditional knockout allele for *Pcgf5* in mice. (A) RT-PCR analysis of *Pcgf5* in BM hematopoietic cell fractions. Cells analyzed were CD34^-^LSK long-term HSCs, CD34^+^Flt3^-^LSK short-term HSCs, CD34^+^Flt3^+^LSK multipotent progenitors (MPPs), common myeloid progenitors (CMPs), granulocyte-macrophage progenitors (GMPs), megakaryocyte-erythroid progenitors (MEPs), and lineage marker^+^ mature hematopoietic cells. *Hypoxanthine-guanosine phosphoribosyl transferase (Hprt)* was used as a housekeeping control gene. Data are shown as the mean ± standard deviation (SD) for triplicate analyses. (B) Strategy for making a conditional knockout allele for *Pcgf5* by homologous recombination in ES cells. FRT recombinase was used to remove the *Neo* cassette. (C) Scheme of the hematopoietic repopulation assay. Total BM cells (5x10^6^ cells) from *Cre-ERT* and *Cre-ERT;Pcgf5*^*fl/fl*^ were transplanted into lethally irradiated CD45.1 recipient mice without competitor BM cells, or 2x10^6^ total BM cells were transplanted with the same number of competitor BM cells. To induce deletion of *Pcgf5*, 100 μl of tamoxifen (10 mg/ml) was intraperitoneally injected once a day for five consecutive days at 1 month post-transplantation. (D) Efficient deletion of *Pcgf5* in hematopoietic cells detected by genomic PCR. Deletion of *Pcgf5* in *Cre-ERT;Pcgf5*^*fl/fl*^ PB myeloid cells was evaluated pre- and post-tamoxifen treatment. “WT”, “Floxed”, and “*Δ*” alleles indicate the wild-type and floxed *Pcgf5* allele, and floxed *Pcgf5* allele after removal of exon 2 by Cre recombinase, respectively. (E) Detection of truncated *Pcgf5* mRNA in BM *Pcgf5*^*Δ/Δ*^ LK cells using primers directed to exon 1 and exon 5/6 junction. (F) Pcgf5 interacts with Ring1B. Ring1B in lysates from *Pcgf5*^*fl/fl*^ and *Pcgf5*^*Δ/Δ*^ ES cells was immunoprecipitated using anti-Ring1b antibody, and then immunoprecipitates were detected by immunoblotting using anti-Ring1b and anti-Pcgf5 antibodies.

We first analyzed the expression of *Pcgf5* in hematopoietic cells by RT-PCR. *Pcgf5* appeared to be preferentially expressed in CD34^-^ Flt3^-^ Lineage marker^-^ Sca-1^+^ c-Kit^+^ (CD34^-^LSK) long-term (LT)-HSCs, CD34^+^Flt3^-^LSK short-term (ST)-HSCs and CD34^+^Flt3^+^LSK multipotent progenitor cells (MPPs), but downregulated during differentiation (Fig 1A). To understand the role of Pcgf5 in hematopoietic stem and progenitor cells (HSPCs), we generated mice harboring *Pcgf5*^*fl*^ allele in which exon 2 containing the first ATG is floxed (Fig 1B) and then crossed *Pcgf5*^*fl/fl*^ mice with *Rosa26*::*Cre-ERT* (*Cre-ERT*) mice to generate *Cre-ERT;Pcgf5*^*fl/fl*^ mice.

To exclude any influences of the loss of *Pcgf5* on organs other than hematopoietic system, we transplanted BM cells from *Cre-ERT* control and *Cre-ERT;Pcgf5*^*fl/fl*^ mice with and without competitor cells into lethally irradiated recipient mice (CD45.1) and deleted *Pcgf5* by intraperitoneal injection of tamoxifen at 1 month post-transplantation (Fig 1C). We confirmed the efficient deletion of *Pcgf5* by genomic PCR of CD45.2 donor cells in the PB (Fig 1D). We also confirmed the generation of a short form of *Pcgf5* mRNA lacking exon 2 in BM Lineage marker^-^ c-Kit^+^ (LK) progenitor cells after injection of tamoxifen (Fig 1E). Pcgf5 functions as a component of PRC1.5. In order to examine whether the functional Pcgf5 proteins are translated from the internal ATG of the short form of *Pcgf5* mRNA, we prepared lysates from *Pcgf5*^*fl/fl*^ and *Pcgf5*^*Δ/Δ*^ ES cells, and immunoprecipitated Ring1b. Pcgf5 was readily detected in the immunoprecipitates from *Pcgf5*^*fl/fl*^ cells, but not *Pcgf5*^*Δ/Δ*^ cells. Even the short form of Pcgf5 was not detected in immunoprecipitates from *Pcgf5*^*Δ/Δ*^ ES cells. These results indicate that no functional Pcgf5 protein that can bind Ring1b is translated from the truncated *Pcgf5* mRNA lacking the first ATG (Fig 1F). The level of Ring1b protein did not largely change in *Pcgf5*^*Δ/Δ*^ hematopoietic cells and ES cells (data not shown).

### Deletion of *Pcgf5* in adult hematopoietic cells does not compromise hematopoiesis

**Fig 2:**
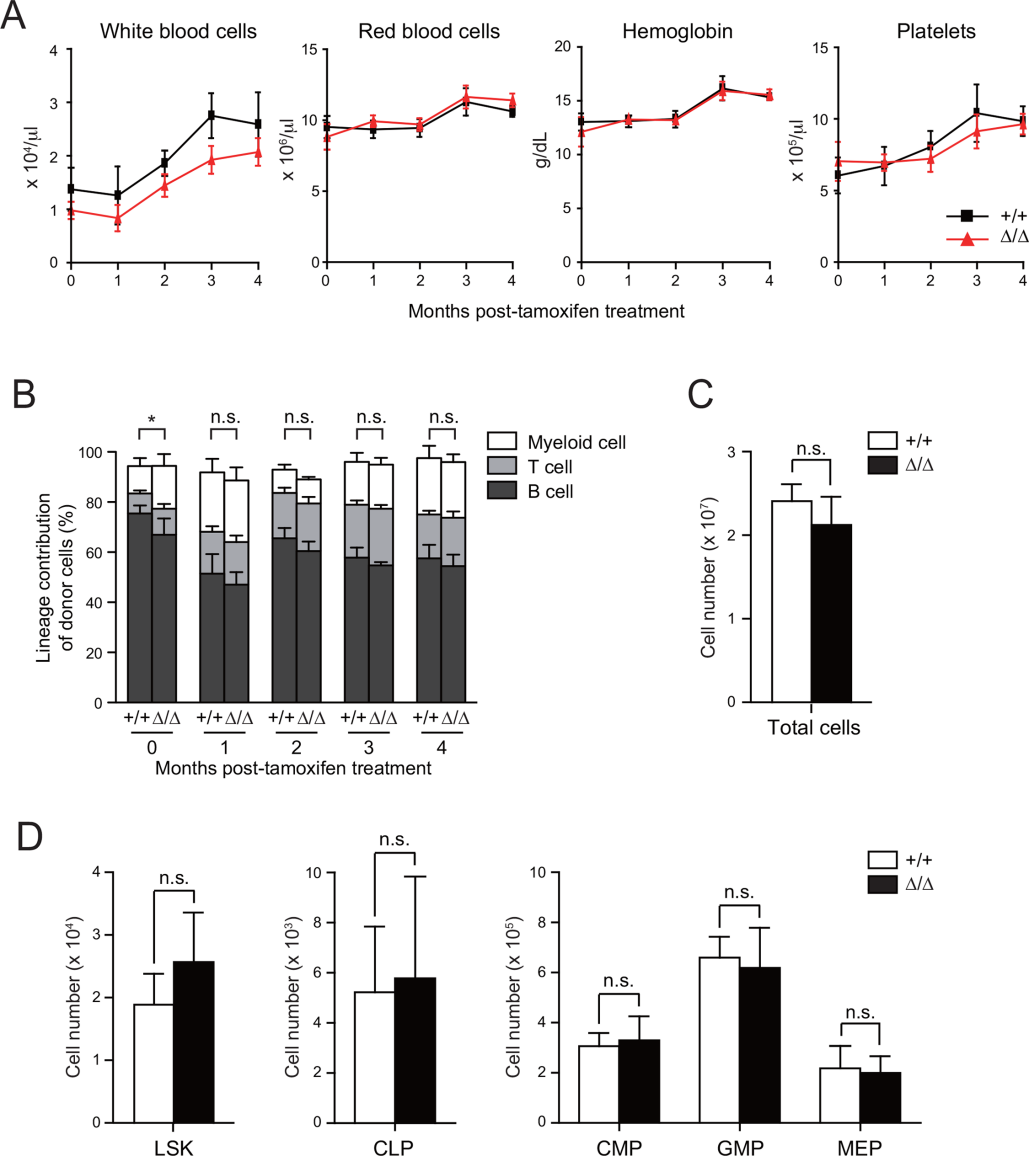
Depletion of *Pcgf5* does not compromise adult hematopoiesis. (A) PB cell counts of recipients repopulated with *Cre-ERT* (+/+) and *Cre-ERT;Pcgf5*^*fl/fl*^ BM cells after deletion of *Pcgf5* (*Δ/Δ*) by tamoxifen injection. Data are shown as mean ± SD (n = 4–5). (B) Lineage contribution of donor cells to myeloid (Gr-1^+^ and/or Mac-1^+^), B (B220^+^), or T (CD4^+^ and/or CD8^+^) cells in the PB shown as mean ± SD (n = 4–5). (C) Absolute number of CD45.2^+^ donor-derived hematopoietic cells in a unilateral pair of femur and tibia of recipients at 5 months post-transplantation. Data are shown as mean ± SD (WT, n = 5; *Pcgf5*^*Δ/Δ*^, n = 6). (D) Absolute number of CD45.2^+^ donor-derived LSK cells, CLPs and myeloid progenitors in the BM of recipient mice at 5 months post-transplantation presented as mean ± SD (WT, n = 5; *Pcgf5*^*Δ/Δ*^, n = 6). n.s., not significant.

In order to evaluate the role of Pcgf5 in HSPCs, we first transplanted BM cells from *Cre-ERT* control and *Cre-ERT;Pcgf5*^*fl/fl*^ mice without competitor cells into lethally irradiated recipient mice. After the deletion of *Pcgf5*, PB cell counts showed moderate reduction in white blood cell (WBC) counts in *Pcgf5*^*Δ/Δ*^ mice, although it did not reach statistical significant levels (Fig 2A). RBC counts, hemoglobin content and platelet counts did not significantly change in the absence of Pcgf5 (Fig 2A). In addition, no obvious change of lineage contribution of donor cells to PB hematopoietic cells was detected after the deletion of *Pcgf5* (Fig 2B). Correspondingly, BM analysis at 4 months post-deletion of *Pcgf5* revealed no significant changes in the number of total BM cells (Fig 2C), LSK HSPCs, common lymphoid progenitors (CLPs) and myeloid progenitors including common myeloid progenitors (CMPs), granulocyte-macrophage progenitors (GMPs), and megakaryocyte-erythroid progenitors (MEPs) (Fig 2D).

**Fig 3:**
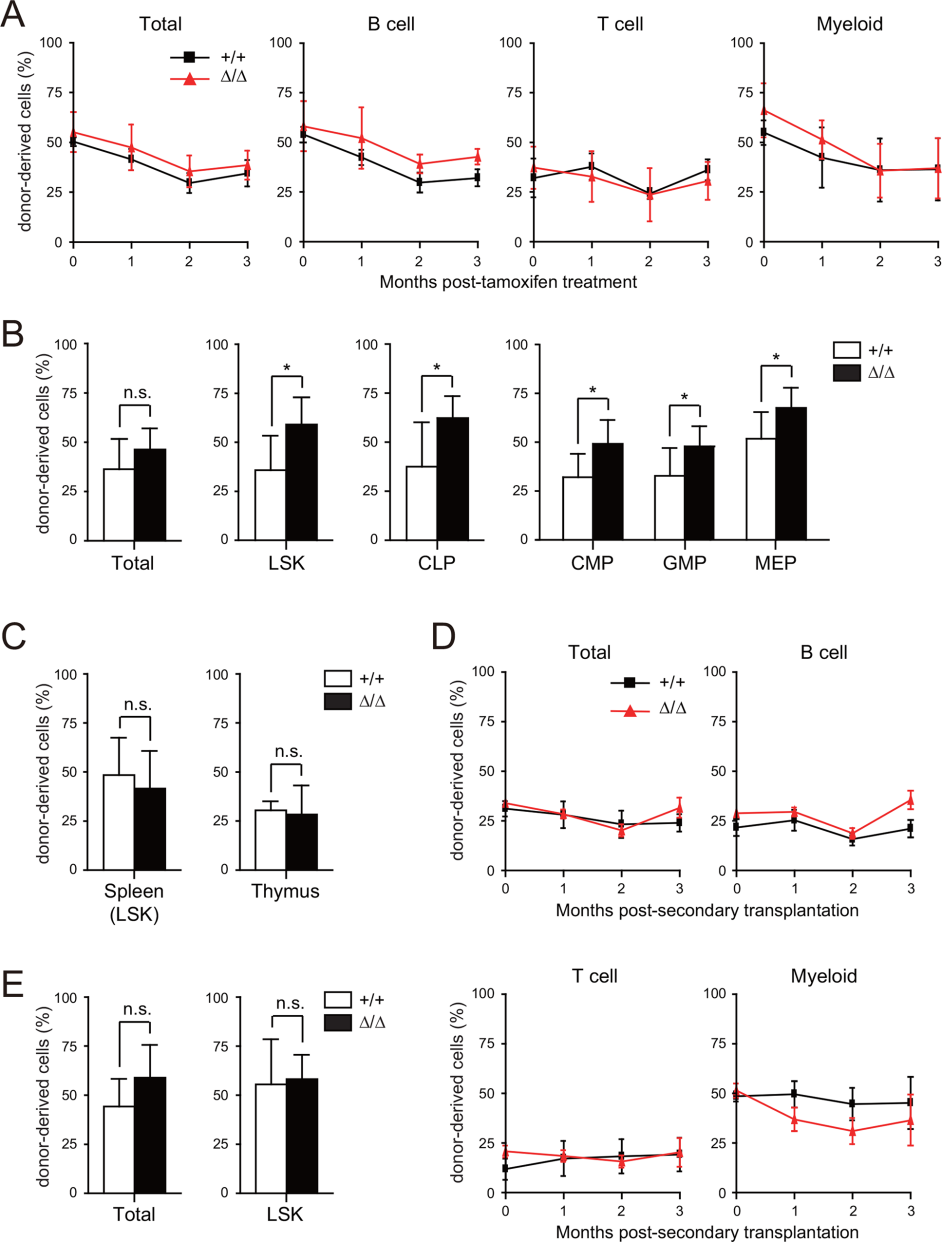
*Pcgf5*-deficient HSPCs retain normal reconstitution capacity of hematopoiesis. (A) Chimerism of donor-derived cells in recipients in competitive reconstitution assays using the same number of test cells (*Cre-ERT* and *Cre-ERT;Pcgf5*^*fl/fl*^) and competitor cells. After engraftment, *Pcgf5* was deleted (*Pcgf5*^*Δ/Δ*^) by tamoxifen injection. Data are shown as mean ± SD (n = 4–5). (B) Chimerism of donor-derived CD45.2^+^ hematopoietic cells in total BM cells, LSK cells, CLPs and myeloid progenitor fractions at 4 months post-transplantation. The data are shown as mean ± SD (n = 7). (C) Chimerism of donor-derived CD45.2^+^ cells in splenic LSK cells and donor-derived CD45.2^+^ thymocytes in the thymus at 4 months post-transplantation shown as mean ± SD (n = 3). (D, E) Secondary transplantation assays. Total BM cells (5x10^6^) from primary recipient mice at 4 months post-transplantation were transplanted into lethally irradiated secondary recipient mice without competitor cells. Chimerism of donor-derived cells in the PB (D) and total CD45.2^+^ hematopoietic cells and LSK cells in the BM (E) at 5 months post-transplantation are shown as mean ± SD (WT, n = 4; *Pcgf5*^*Δ/Δ*^, n = 5). *p<0.05; n.s., not significant.

We next explored the consequences of *Pcgf5* loss on the competitive repopulating capacity of HSPCs. We transplanted BM cells from *Cre-ERT* control and *Cre-ERT;Pcgf5*^*fl/fl*^ mice with the same number of competitor cells from CD45.1 congenic wild-type (WT) mice into lethally irradiated recipient mice. Even in this competitive setting, no evident changes were detected in chimerism of CD45.2^+^ *Pcgf5*^*Δ/Δ*^ cells in the PB compared with the control (Fig 3A). BM analysis at 3 months post-deletion of *Pcgf5* revealed a mild but significant increase in the chimerism of CD45.2^+^ *Pcgf5*^*Δ/Δ*^ cells in total cells, LSK HSPCs, CLPs, and myeloid progenitors in the BM (Fig 3B), but not in splenic LSK cells nor total thymocytes in the thymus (Fig 3C). To further evaluate the repopulating capacity of *Pcgf5*^*Δ/Δ*^ HSPCs, we analyzed BM from secondary recipients transplanted with pooled BM cells from primary mice. Chimerism of *Pcgf5*^*Δ/Δ*^ cells was comparable to WT cells in both PB and BM of secondary recipients (Fig 3D and 3E). The trend of *Pcgf5*^*Δ/Δ*^ HSPCs toward higher chimerism in the BM of primary mice totally disappeared in the secondary recipients. These results suggest that the loss of *Pcgf5* does not significantly alter reconstitution capacity of HSPCs.

### Loss of Pcgf5 has a limited effect on the transcriptional profiles of HSPCs

**Fig 4:**
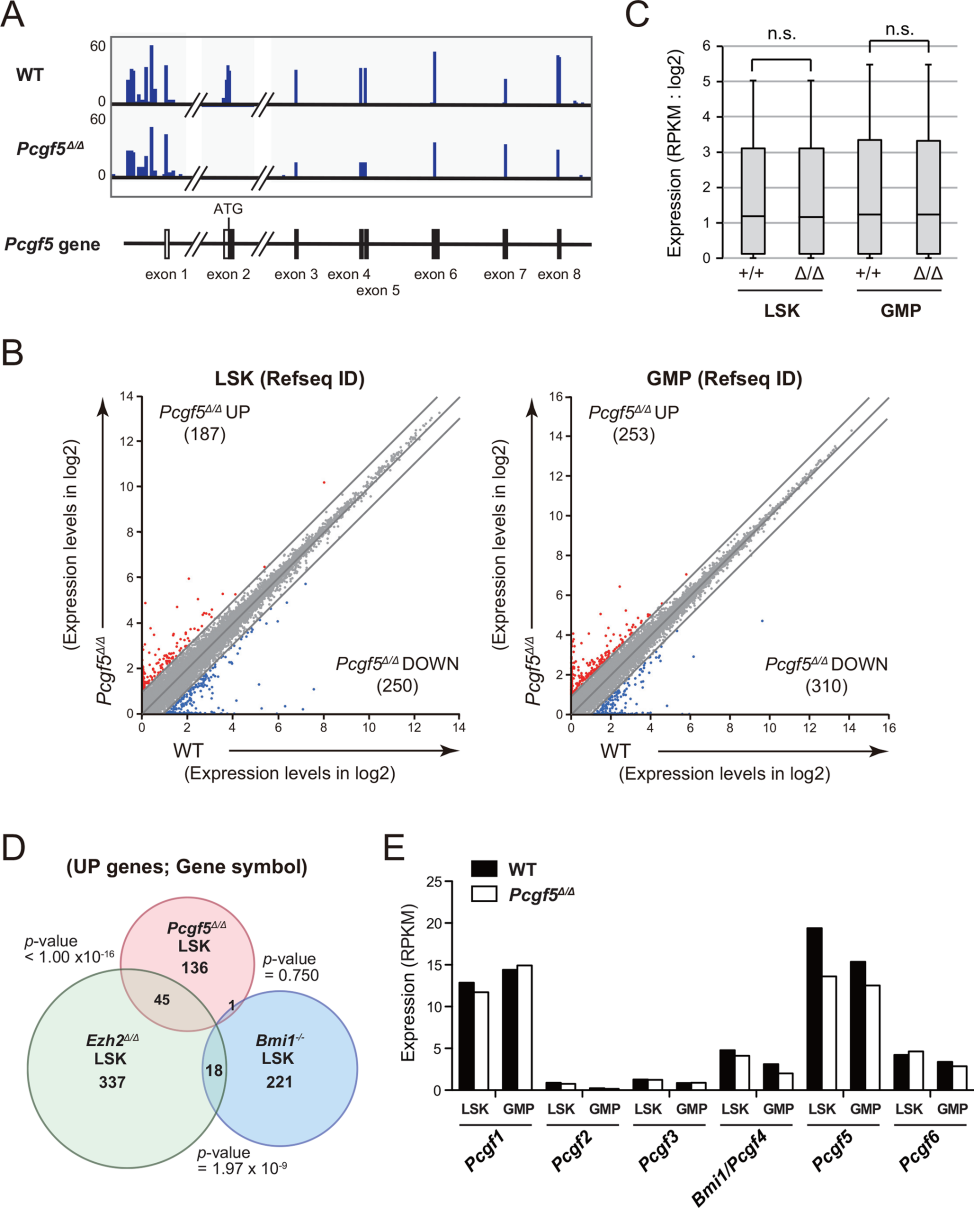
Gene expression profile of *Pcgf5*-deficient HSPCs. (A) Snapshots of RNA-sequence signals at the *Pcgf5* gene locus in WT and *Pcgf5*^*Δ/Δ*^ LSK cells isolated from recipient mice repopulated with *Pcgf5*^*Δ/Δ*^ hematopoietic cells. The structure of *Pcgf5* gene locus including relevant exons is indicated at the bottom. (B) Scatter diagrams showing RNA-sequence data of LSK cells and GMPs. Expression levels of RefSeq genes (listed in RefSeq ID) defined by reads per kilobase of exon per million fragments mapped (RPKM) in log2 in WT and *Pcgf5*^*Δ/Δ*^ cells are plotted. The light gray lines represent the boundaries for twofold increase and twofold decrease, respectively. The number of genes upregulated and downregulated more than twofold in *Pcgf5*^*Δ/Δ*^ cells compared with WT cells are indicated in red and blue, respectively. (C) Box-and-whisker plots showing the expression levels of WT and *Pcgf5*^*Δ/Δ*^ LSK cells and GMPs in RPKM. Boxes represent 25–75 percentile ranges. Vertical lines represent 10–90 percentile ranges. Horizontal bars represent medians. n.s., not significant. (D) Venn diagram showing the overlap between genes (listed in gene symbol) up-regulated in LSK cells from *Pcgf5*^*Δ/Δ*^, *Ezh2*^*Δ/Δ*^ [18], and *Bmi1* KO [19] mice (≥2.0-fold compared with the WT control, respectively). The numbers of genes in each group are indicated. The statistical significance of the overlaps between the two gene groups is indicated. (E) Expression of *Pcgf* genes in WT LSK cells and GMPs in RPKM.

To understand the effects of Pcgf5 loss on HSPCs, we next purified LSK HSPCs and GMPs from BM at 4 months post-deletion of *Pcgf5* and performed RNA-sequence analysis. RNA-sequence data confirmed the absence of *Pcgf5* transcript corresponding to exon 2 deleted in *Pcgf5*^*Δ/Δ*^ cells (Fig 4A). The number of genes altered greater than 2-fold in the absence of Pcgf5 was relatively small (Fig 4B) and the expression changes were mild (Fig 4B). Indeed, the levels of expression changes in *Pcgf5*^*Δ/Δ*^ cells were not statistically significant compared with WT cells (Fig 4C). Genes upregulated greater that 2-fold in *Pcgf5*^*Δ/Δ*^ LSK cells significantly overlapped with those in *Ezh2*^*Δ/Δ*^ LSK cells [18], but, of interest, barely with those in *Pcgf4/Bmi1*^*Δ/Δ*^ LSK cells [10] (Fig 4D). Given the minimal hematological phenotypes in the absence of Pcgf5, we assumed that the other Pcgf family genes were activated to complement Pcgf5 loss. However, RNA-seq data did not show activation of any other family genes in the absence of Pcgf5 (Fig 4E). Nevertheless, RNA-sequence data revealed that the expression of *Pcgf1* and *Pcgf5* in Reads Per Kilobase of exon per Million mapped fragments (RPKM) was much higher than the other member genes in LSK cells and GMPs (Fig 4E), suggesting that *Pcgf1* and *Pcgf5* are the major Pcgf family genes expressed in HSPCs.

### Global levels of H2AK119ub1 are significantly reduced in the absence of Pcgf5

**Fig 5:**
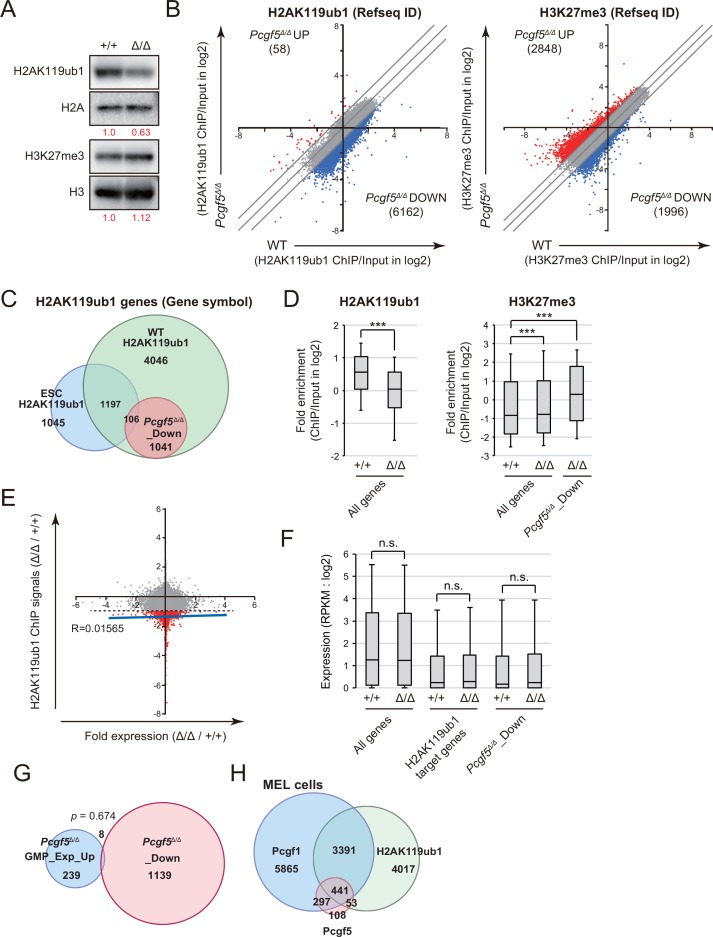
Loss of Pcgf5 results in reduction in global H2AK119 levels. (A) H2AK119ub1 levels in LK cells. LK cells from BM of WT and *Pcgf5*^*Δ/Δ*^ mice were analyzed by Western blotting using anti-H2AK119ub1 and anti-H3K27me3 antibodies at 1-month post deletion of *Pcgf5*. Levels of H2AK119ub1 and H3K27me3 were normalized to the amount of H2A and H3, respectively, and are indicated relative to wild-type control values at the bottom. The representative data from two independent experiments are presented. (B) Scatter plots showing the correlation of the fold enrichment values against the input signals (ChIP/input) (transcription start site ± 2.0 kb) of H2AK119ub1 and H3K27me3 of RefSeq genes (listed in RefSeq ID) between WT and *Pcgf5*^*Δ/Δ*^ GMPs. The light gray lines represent the boundaries for twofold increase and twofold decrease, respectively. The changes of H2AK119ub1 or H3K27me3 levels in genes upregulated and downregulated more than twofold in *Pcgf5*^*Δ/Δ*^ cells compared with WT cells are indicated in blue and red, respectively. (C) Venn diagram showing the overlap between H2AK119ub1 genes (listed in gene symbol) in GMPs and ES cells. *Pcgf5*^*Δ/Δ*^ _Down genes are also depicted. (D) Box-and-whisker plots showing H2AK119ub1 and H3K27me3 levels in all Refseq genes (All genes) and H2AK119ub1 genes (genes with ≥ 2-fold enrichment of H2AK119ub1 ChIP signals over the input signals at 2.0 kb ± TSSs in WT GMPs) which showed reduction in H2AK119ub1 levels ≥ 2-fold in *Pcgf5*^*Δ/Δ*^ GMPs (*Pcgf5*^*Δ/Δ*^_Down genes). ***p <0.001 (Student *t* test). (E) Scatter plots showing the correlation of the fold expression and fold enrichment of H2AK119ub1 ChIP signals in *Pcgf5*^*Δ/Δ*^ GMPs compared with those in WT GMPs. The genes showing reduction in H2AK119ub1 levels greater than 2-fold (below dotted line) are indicated in red dots. The score of correlation coefficient between the fold expression and fold enrichment of H2AK119ub1 ChIP signals defined with Pearson’s correlation and the linear regression are shown. (F) Box-and-whisker plots showing the expression levels of all RefSeq genes, H2AK119ub1 genes, and *Pcgf5*^*Δ/Δ*^_Down genes in WT and *Pcgf5*^*Δ/Δ*^ GMPs in RPKM. Boxes represent 25–75 percentile ranges. Vertical lines represent 10–90 percentile ranges. Horizontal bars represent medians. n.s., not significant. (G) Venn diagram showing the overlap between *Pcgf5*^*Δ/Δ*^_Down genes (listed in gene symbol) in GMPs and genes upregulated in expression greater than 2-fold in *Pcgf5*^*Δ/Δ*^ GMPs relative to WT GMPs. (H) ChIP-sequence data of 3xFlag-Pcgf1, 3xFlag-Pcgf5, and H2AK119ub1 in MEL cells. Venn diagram showing the overlap between Pcgf1 targets, Pcgf5 targets, and H2AK119ub1 genes (≥ 2-fold enrichment of ChIP signals over the input signals at 2.0 kb ± TSSs) (listed in gene symbol) in MEL cells.

As shown in Fig 1F, Pcgf5 functions as a component of PRC1-related complex. As expected from these data, global H2AK119ub1 level was decreased by 40% in *Pcgf5*^*Δ/Δ*^ Lin^-^c-Kit^+^ progenitor cells in Western blot analysis, while H3K27 level was not altered in the absence of Pcgf5 (Fig 5A).

We next performed ChIP-sequence analysis of H2AK119ub1 and H3K27me3 in GMPs from recipient mice at 4 months post-deletion of *Pcgf5*. We defined genes with ≥ 2-fold enrichment of H2AK119ub1 ChIP signals over the input signals at the promoter region (2.0 kb ± transcriptional start sites) as H2AK119ub1 genes (Fig 5B). H2AK119ub1 genes in WT GMPs significantly overlapped with genes marked with H2AK119ub1 in ES cells [19] (Fig 5C). Importantly, nearly 20% of H2AK119ub1 genes showed reduction in H2AK119ub1 levels ≥ 2-fold upon loss of Pcgf5 in GMPs (Fig 5B and 5C). Indeed, H2AK119ub1 ChIP signals over the input signals were significantly reduced in *Pcgf5*^*Δ/Δ*^ GMPs compared with WT GMPs, while those of H3K27me3 showed a very mild albeit significant increase in *Pcgf5*^*Δ/Δ*^ GMPs (Fig 5D). Polycomb-related histone marks, H2AK119ub1 and H3K27me3, mutually reinforce each other and behave in a similar manner in many settings. Unexpectedly, however, H2AK119ub1 genes that showed reduction in H2AK119ub1 levels ≥ 2-fold in *Pcgf5*^*Δ/Δ*^ GMPs (*Pcgf5*^*Δ/Δ*^_Down genes) showed a significant increase in H3K27me3 levels (Fig 5D). In contrast to our expectation, comparison of ChIP signals with expression changes revealed no significant correlation of reduced H2AK119ub1 levels with gene expression (Fig 5E and 5F). Moreover, *Pcgf5*^*Δ/Δ*^_Down genes (listed in gene symbol) little overlapped with genes upregulated greater than 2-fold in *Pcgf5*^*Δ/Δ*^ GMPs relative to WT GMPs (*Pcgf5*^*Δ/Δ*^ GMPs_Exp_Up)(Fig 5G). These findings well correspond to the mild changes in global gene expression and minimal hematological phenotypes in *Pcgf5*^*Δ/Δ*^ HSPCs.

In order to understand the minimal effect of the loss of Pcgf5 on gene expression, we overexpressed 3xFlag-Pcgf1 and 3xFlag-Pcgf5 in mouse erythroleukemia (MEL) cells and performed ChIP-sequence analysis of Pcgf1, Pcgf5 and H2K119ub1. We found that Pcgf1 regulated more gene promoters (2.0 kb ± transcriptional start sites) than Pcgf5 and also bound most of the Pcgf5 targets (82.1%). Among Pcgf5 targets, Pcgf1 regulated the majority of Pcgf5 targets associated with the H2AK119ub1 modification (89.3%) in MEL cells. (Fig 5H and S1 Table). These findings suggest that Pcgf1 largely compensates for the loss of Pcgf5.

## Discussion

In this study, we generated *Pcgf5* conditional knockout mice and found that the hematopoietic-specific deletion of *Pcgf5* results in no significant changes in hematopoiesis compared with control mice. However, Pcgf5 appeared to contribute to the global monoubiqutination of H2AK119 in hematopoietic cells. Although the absence of Pcgf5 did not greatly affect the gene expression profiles of HSPCs, our findings provide the first direct evidence that supports PRC1-related function of Pcgf5 that is involved in the regulation of H2AK119ub1.

Pcgf5 has repeatedly been identified as a component of variant PRC1 that include Auts2 (PRC1.5). As other Pcgf family proteins, Pcgf5 has been thought to support the monoubiquitination of H2AK119 by Ring1b, however, this has never been conformed *in vivo*. In this study, *Pcgf5*-deficient HSPCs clearly showed reduction in H2AK119ub1 levels. Nearly 20% of gene promoters (1,147 genes) marked with H2AK119ub1 in WT GMPs reduced H2AK119ub1 levels greater that 2-fold in GMPs in the absence of Pcgf5. This finding suggests that Pcgf5 targets a large number of genes and plays a major role in the regulation of H2AK119ub1. Upregulated genes in *Pcgf5*-deficient LSK cells significantly overlapped with those in *Ezh2*-deficient LSK cells, suggesting that Pcgf5 targets largely overlap with those of PRC2. In contrast, upregulated genes in *Pcgf5*-deficient LSK cells did not significantly overlap with those in *Pcgf4/Bmi1*-deficient LSK cells, suggesting that Pcgf5 in variant PRC1 regulates genes distinct from those of canonical PRC1 that contains Pcgf4/Bmi1 in HSPCs. Recently, Pcgf2/Mel18-containing PRC1 complexes have been reported to exchange subunits in a stage-specific manner during cardiac differentiation and regulate both transcriptional repression and activation of distinctive sets of target genes [20]. Because more genes were downregulated upon *Pcgf5* deletion, Pcgf5 could function like Pcgf2/Mel18 in a context-specific manner.

As described above, Pcgf5 as well as Pcgf1 and Pcgf3, has been shown to recruit PRC2 in an H2AK119ub1-dependent manner and induce H3K27me3 modification at its target genes in a *de novo* targeting assay in mouse ESCs [17]. Therefore, we expected to see a reduction in H3K27me3 levels at the Pcgf5 target genes. However, H2AK119ub1 genes that showed reduction in H2AK119ub1 levels (≥ 2-fold) in *Pcgf5*^*Δ/Δ*^ GMPs (*Pcgf5*^*Δ/Δ*^_Down genes) displayed rather increased H3K27me3 levels at their promoters. Although the molecular mechanism underlying this epigenomic alteration remains unclear, it is possible that several different pathways that recruit polycomb complexes exist as backup, and some of them could be activated in a setting of polycomb dysfunction. In the case of Pcgf5 loss, we identified augmentation in H3K27me3 levels, which might represent activation of the compensatory pathway and could be responsible for the maintenance of transcriptional repression of the *Pcgf5*^*Δ/Δ*^_down genes in HSPCs.

*Pcgf5* expression is high in HSPCs compared with differentiated cells and appeared to be the major Pcgf family gene expressed in HSPCs. RNA-sequence data demonstrated that *Pcgf1* and *Pcgf5* are most abundant in HSPCs. However, the impact of loss of Pcgf5 was very limited in HSPCs. These findings suggest that Pcgf5 function could be compensated by other family members such as Pcgf1, which is highly expressed in HSPCs. Pcgf1 is the component of the non-canonical PRC1 complex, PRC1.1, and has been demonstrated to cause a drastic reduction in H2AK119ub1 levels in murine ESCs upon knockdown [21]. Indeed, ChIP-sequence analysis in MEL cells demonstrated that Pcgf5 targets are mostly co-regulated by Pcgf1. Given that the loss of Pcgf5 was largely compensated for in HSPCs, the role of Pcgf1-containing PRC1.1 could be more critical than Pcgf5-containing PRC1.5 in the maintenance of HSPCs. Further study of non-canonical PRC1 complexes is needed to decipher their physiological functions in HSPCs.

Although Pcgf5 appears to be dispensable for hematopoiesis, Pcgf family gene expression could vary in different organs and tissues and Pcgf5 may be required for the proliferation, survival and function of certain types of cells. The mice that harbor floxed allele for *Pcgf5* generated in this study should serve as a valuable tool to analyze the role of Pcgf5 in those cells.

## Supporting Information

S1 Table Target genes of Pcgf5, Pcgf1 and H2AK119ub1 in MEL cells identified by ChIP-Seq. (XLSX)
